# Supplementary material for: Sequencing and assembly of the Egyptian buffalo genome
Source: PLoS One. 2020 Aug 19;15(8):e0237087. doi: 10.1371/journal.pone.0237087 (PMC7437910; doi:10.1371/journal.pone.0237087)

**Implementation of the assembly workflow**

The assembly and scaffolding workflow is composed of staged execution of software tools. The workflow is packaged as a docker image (https://hub.docker.com/r/mshokrof/buffalo). The docker image includes a Tavaxy-based implementation. There is also a demo version of the workflow accessible from (<https://tavaxy.org/buffalo> <http://s2.eg-bioinformatics.com/tavaxy/webui/login.html>). The following figure shows screen shot including the different steps of the workflow as implemented in Tavaxy. The use of Tavaxy provides a visual interface to the workflow and the users can see the parameters and the sequence of tasks to be implemented. The Tavaxy implementation is useful at the stage of design and testing the pipeline, where the parameters have to be tuned. To avoid the complexities of installation, the docker image includes all dependency to run Tavaxy and the analysis. The image also includes all binaries in case the user likes to run individual steps using command line interface.

**Tavaxy Implementation**

The following figure shows the workflow used for assembling the buffalo genome based on SOLiD sequencing. The workflow is modeled as a graph where the nodes are the steps to be excuted using the respective tools. The edges represent dependencies and data transferred from one node to the next for subsequent execution. The zoom-in boxes show the details of the mapping and assembly steps. The left pane of Tavaxy includes list of tools. The parameters of each step/node can be adjusting by clicking on each tool box.

**
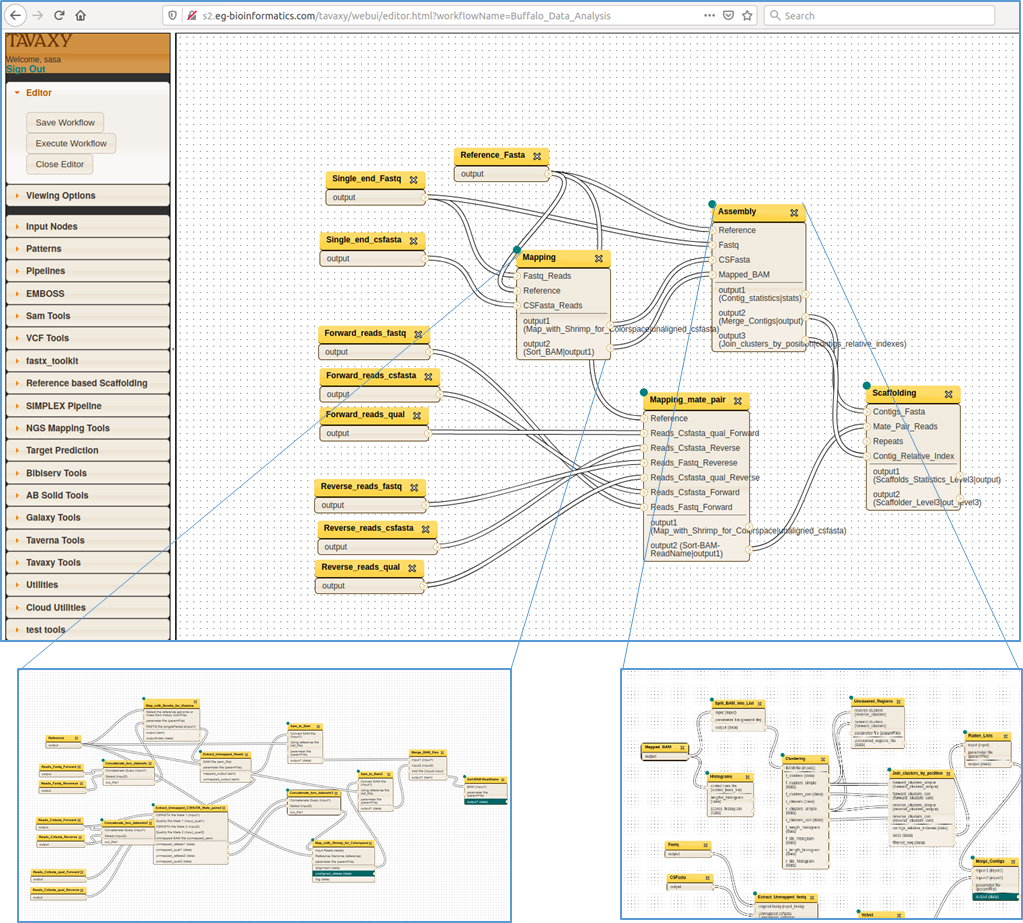
**

**Steps to use Tavaxy Docker container**

1. sudo docker run -it -P -p 80:80 -p 8306:3306 --name tavaxy mshokrof/buffalo
2. On the docker container run:
   1. service apache2 start
   2. service mysql start
   3. /var/www/tavaxy/engines/galaxy/run.sh > ~/out 2> ~/err &
3. Open on the browser url: ip-address/tavaxy/webui/index.html
4. Log in with user: sasa and pass: 123
5. Click on My Workflows(Second item in the middle)
6. Click Execute on Buffalo Data analysis
7. Fill the input as follows
8. Run the workflow by clicking on the run button


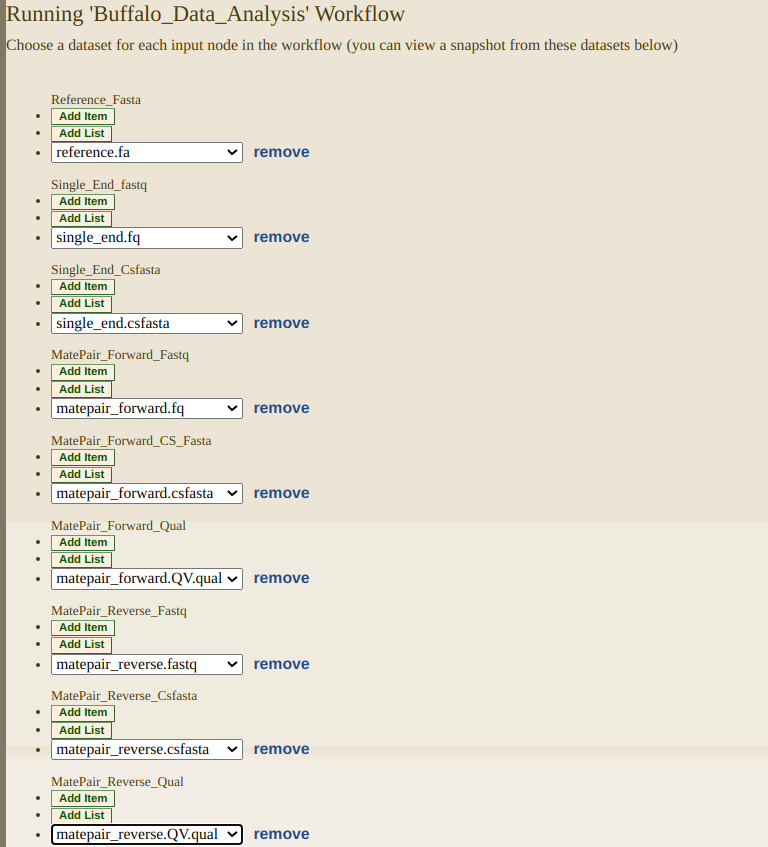


For more Information, plz visit the project web-site

[**https://buffalo.tavaxy.org/**](https://buffalo.tavaxy.org/)

**
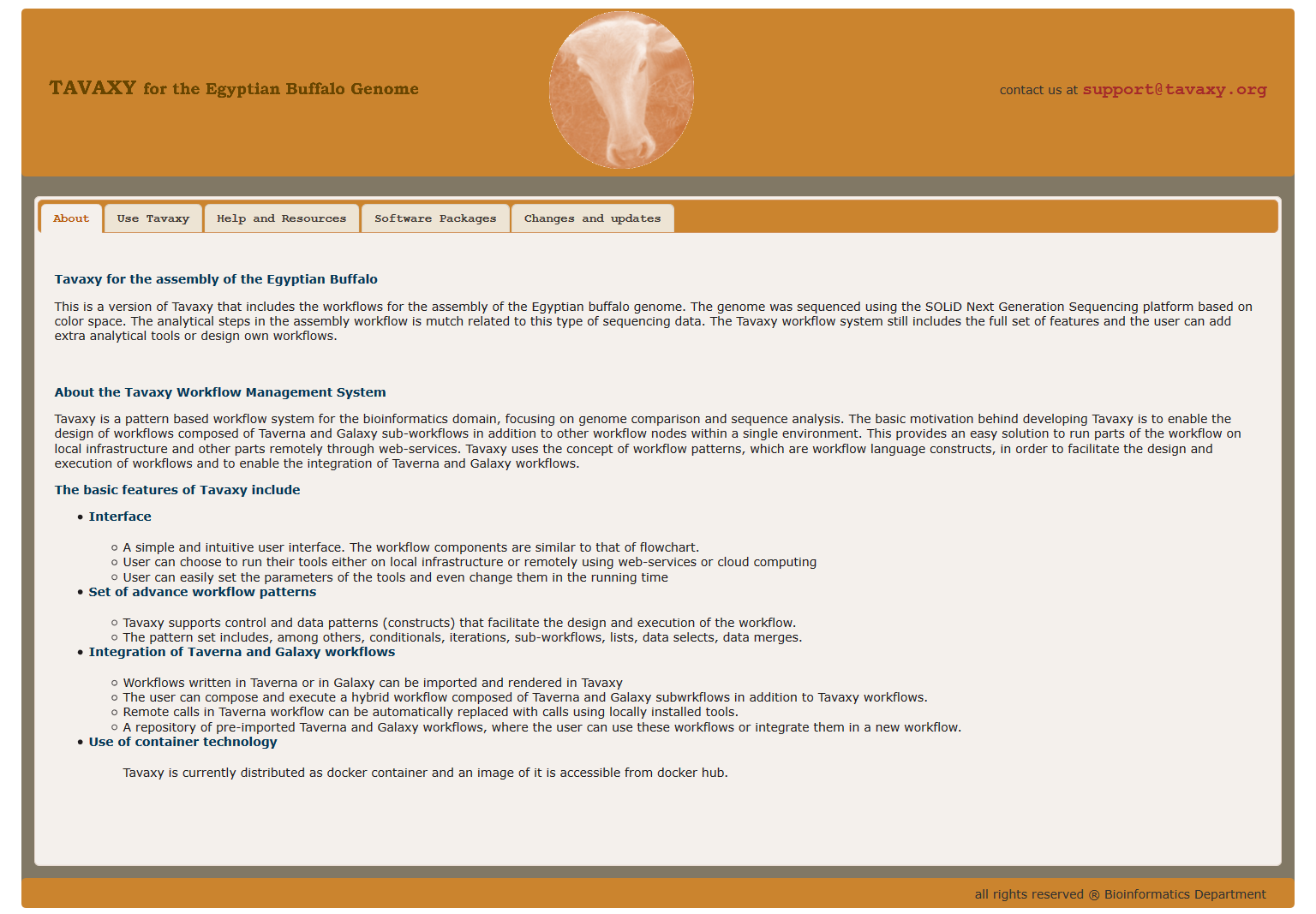
**

**For using the demo version for small data, plz go the tab “Use Tavaxy”.**


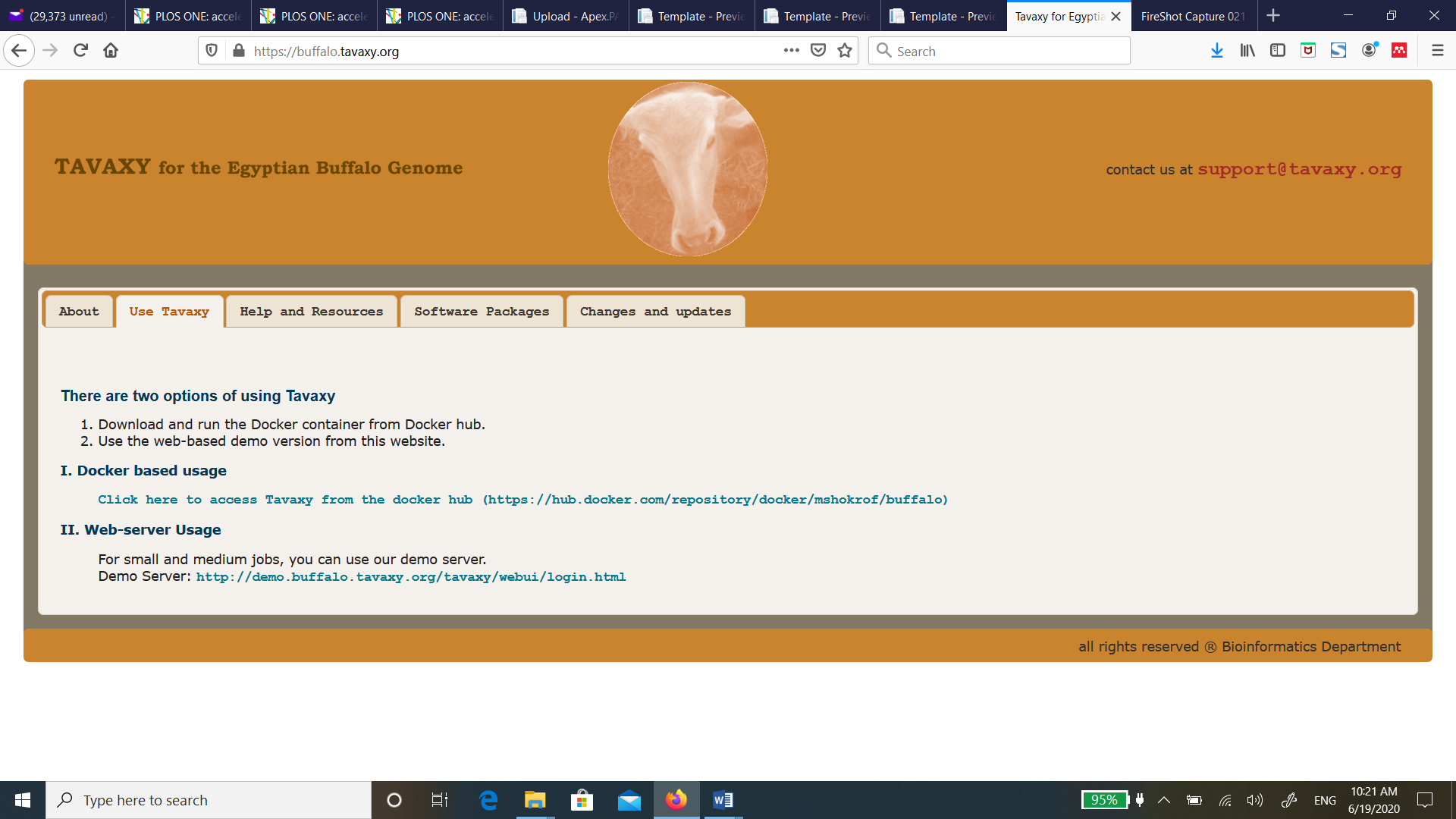


**For extra information, plz access the documentation at the help tab..**


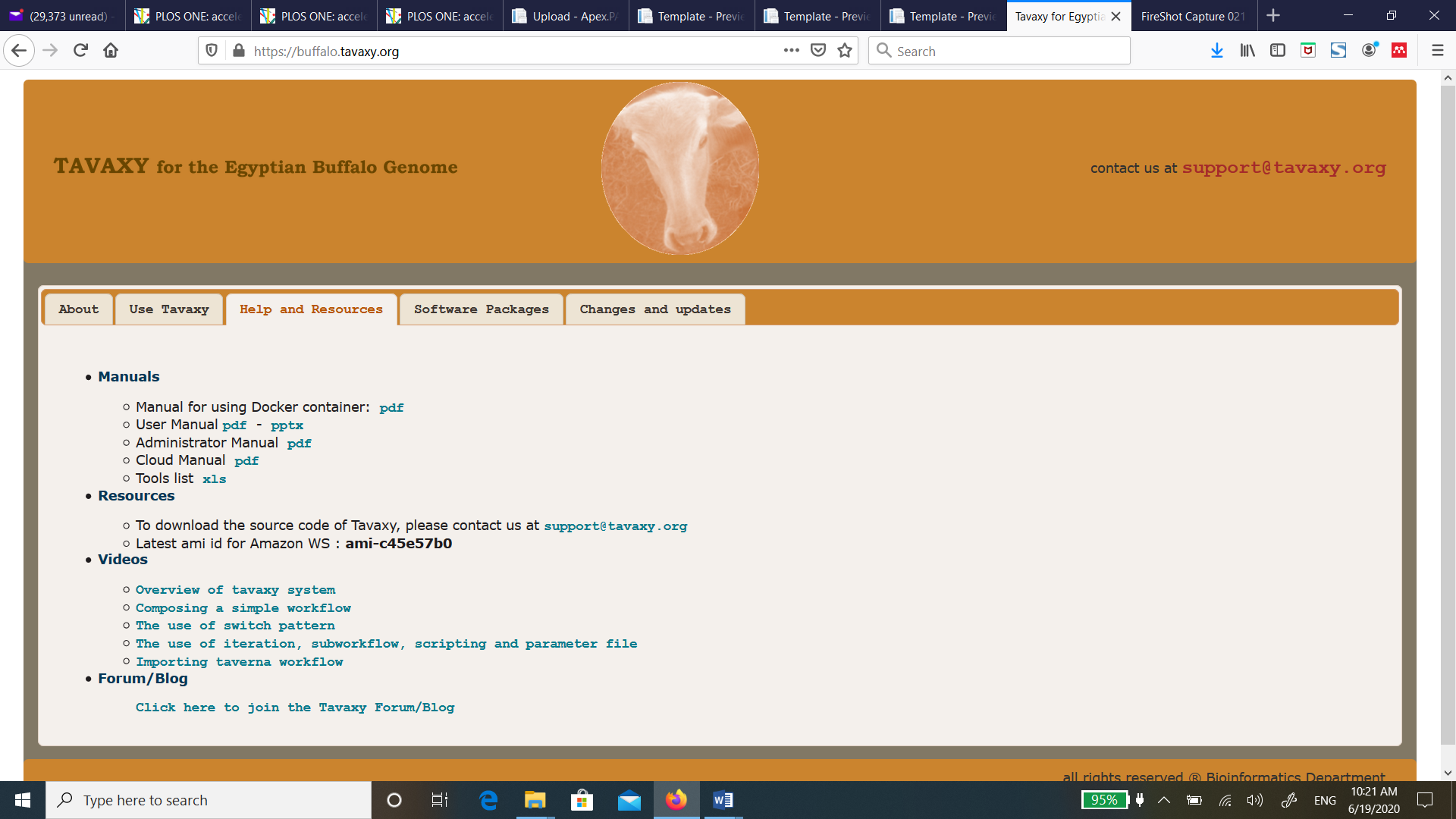

Supplement: S1 File — (DOCX) [file pone.0237087.s001.docx]
