# Supplementary material for: Sequencing and assembly of the Egyptian buffalo genome
Source: PLoS One. 2020 Aug 19;15(8):e0237087. doi: 10.1371/journal.pone.0237087 (PMC7437910; doi:10.1371/journal.pone.0237087)
Supplement: S2 File — (DOCX) [file pone.0237087.s002.docx]

| CattleChr | Length Cattle Chr. | %Cov (Reads) | %Cov (Contigs) | %Repeat |
| --- | --- | --- | --- | --- |
| Chr1 | 158337067 | 98.53% | 98.21% | 49.37 |
| Chr2 | 137060424 | 98.66% | 98.35% | 47.69 |
| Chr3 | 121430405 | 98.4% | 97.9% | 49.17 |
| Chr4 | 120829699 | 98.5% | 98.11% | 46.81 |
| Chr5 | 121191424 | 98.24% | 97.64% | 48.11 |
| Chr6 | 119458736 | 98.23% | 97.68% | 49.82 |
| Chr7 | 112638659 | 97.23% | 96.53% | 47.41 |
| Chr8 | 113384836 | 98.24% | 97.71% | 49.12 |
| Chr9 | 105708250 | 98.34% | 97.89% | 47.52 |
| Chr10 | 104305016 | 98.7% | 98.34% | 46.82 |
| Chr11 | 107310763 | 98.27% | 97.67% | 45.75 |
| Chr12 | 91163125 | 98.38% | 97.95% | 47.29 |
| Chr13 | 84240350 | 98.46% | 97.86% | 44.9 |
| Chr14 | 84648390 | 98.42% | 97.87% | 46.18 |
| Chr15 | 85296676 | 98.08% | 97.5% | 49.96 |
| Chr16 | 81724687 | 98.21% | 97.63% | 47.08 |
| Chr17 | 75158596 | 97.37% | 96.06% | 48.05 |
| Chr18 | 66004023 | 98.02% | 97.14% | 44.54 |
| Chr19 | 64057457 | 97.78% | 96.8% | 42.09 |
| Chr20 | 72042655 | 98.575 | 98.19% | 47.25 |
| Chr21 | 71599096 | 98.17% | 97.41% | 48.01 |
| Chr22 | 61435874 | 98.34% | 97.63% | 43.75 |
| Chr23 | 52530062 | 98.32% | 97.76% | 42.89 |
| Chr24 | 62714930 | 98.6% | 98.22% | 44.29 |
| Chr25 | 42904170 | 97.65% | 96.21% | 45.42 |
| Chr26 | 51681464 | 98.28% | 97.7% | 44.64 |
| Chr27 | 45407902 | 97.4% | 96.61% | 44.76 |
| Chr28 | 46312546 | 98.32% | 97.86% | 48.34 |
| Chr29 | 51505224 | 97.53% | 96.45% | 46.83 |
| ChrX | 148823899 | 98.5% | 95.8% | 56.61 |
| **Totals/Averages** |  | **98.1%** | **97.5%** | **47%** |

**Supplementary Table 2: Buffalo Reads and Contigs Mapping against the Cattle Genome (Detailed View per Cattle chromosomes)**
